# Radiological Biomarkers for Brain Metastases Prognosis: Quantitative Magnetic Resonance Imaging (MRI) Modalities As Non-invasive Biomarkers for the Effect of Radiotherapy

**DOI:** 10.7759/cureus.38353

**Published:** 2023-04-30

**Authors:** Akram M Eraky

**Affiliations:** 1 Neurological Surgery, Medical College of Wisconsin, Milwaukee, USA

**Keywords:** quantitative imaging, brain tumors (primary or brain metastasis), radiologic biomarkers, miliary brain metastasis, magnetic-resonance imaging (mri), solitary brain tumor metastases (sbms), arterial spin labelling (asl), dynamic contrast enhanced mri (dce-mri), dsc mri, diffusion weighted imaging (dwi)

## Abstract

Radiotherapy effect is achieved by its ability to cause DNA damage and induce apoptosis. In contrast, radiation can induce tumor cells’ proliferation, invasiveness, and epithelial-mesenchymal transition (EMT). Besides developing radioresistance, this paradoxical effect of radiotherapy is considered a challenging problem in the field of radiotherapy. This highlights the importance of developing new modalities to diagnose radioresistance early to avoid any unnecessary exposure to radiation and differentiate between metastases recurrence versus post-radiation changes. Quantitative magnetic resonance imaging (MRI) techniques including diffusion-weighted imaging (DWI), dynamic susceptibility contrast (DSC), arterial spin labeling (ASL), and dynamic contrast-enhanced (DCE) represent potential biomarkers to diagnose metastases recurrence and radioresistance. In this review, we will focus on recent studies discussing the possibility of using DWI, DSC, ASL, and DCE to diagnose radioresistance and recurrence in patients with brain metastases.

## Introduction and background

**Figure 1:**
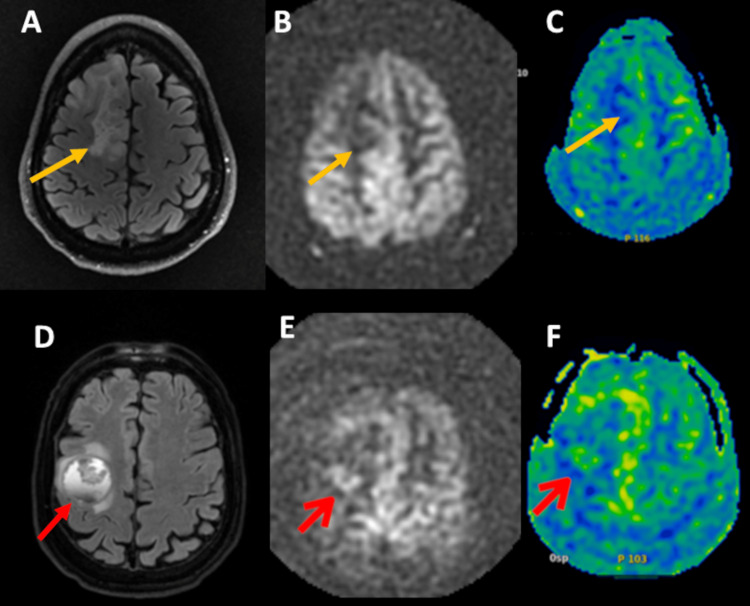
Role of ASL MRI in differentiating between different brain tumors. (A, B, C) The images represent a right frontal lobe oligodendroglioma (orange arrow). (D, E, F) The images represent right frontal lobe non-small cell lung cancer metastasis (red arrow). (A) FLAIR MRI shows a right frontal lobe oligodendroglioma. (B, C) axial pcASL (B) and color-coded CBF map (C) from pcASL data show no hyperperfusion. (D) FLAIR MRI shows a right frontal lobe non-small cell lung cancer metastasis with perilesional vasogenic edema. (E, F) axial pcASL (E) and color-coded CBF map (F) from pcASL data show heterogenous hyperperfusion amongst nodules at the super lesion margin. FLAIR, fluid attenuated inversion recovery; MRI, magnetic resonance imaging; ASL, arterial spin labeling; pcASL, pseudo-continuous arterial spin labeling; CBF, cerebral blood flow

Brain metastases (MTs) are the most common brain tumors. Brain MTs arise from primary tumors from distant organs, such as the lung, kidney, prostate, breast, and colon. Brain MTs are found to be difficult to diagnose especially if they present as a solitary mass without systemic manifestations [1]. Now the only available modality to diagnose brain MT and differentiate between MT recurrence and post-radiation changes is the histopathologic examination of the tumor through biopsy. Having non-invasive rapid biomarkers to diagnose brain MTs is essential to help in early diagnosis and avoid invasive biopsy and unnecessary radiation [2-4]. Using advanced MRI techniques as radiologic biomarkers become promising, non-invasive modalities for brain MTs diagnosis, MT recurrence identification, and radioresistance recognition. In contrast to conventional magnetic resonance imaging (MRI), which provides information about the anatomical features of the brain lesion of interest, quantitative MRI techniques provide information about the physiologic features of the brain lesion, such as vascularity, permeability, metabolism, and cellularity [5,6]. The term quantitative MRI in our article includes diffusion-weighted imaging (DWI) and perfusion-weighted imaging (PWI) which includes dynamic susceptibility contrast (DSC) MRI, dynamic contrast-enhanced (DCE) MRI, and arterial spin labeling (ASL) MRI [7-10]. These advanced techniques can provide us with qualitative, semi-quantitative, and quantitative parameters. ASL labels magnetically the water molecules in the cerebral blood vessels and uses these water molecules as endogenous tracers to visualize and quantify the cerebral blood perfusion (Figure 1) [7,11,12]. In Figure 1, we present two different brain tumors having different blood perfusion on ASL. This demonstrates that in contrast to conventional MRI which shows the anatomical features and gross changes in the lesion of interest, such as necrosis, hemorrhage, and cystic changes, ASL as one of the advanced MRI techniques can depict the physiologic features of the lesion of interest. This also shows the potential role of ASL in differentiating between different tumors by exposing their different physiologic features, such as permeability and vascularity.​​​

**Figure 2:**
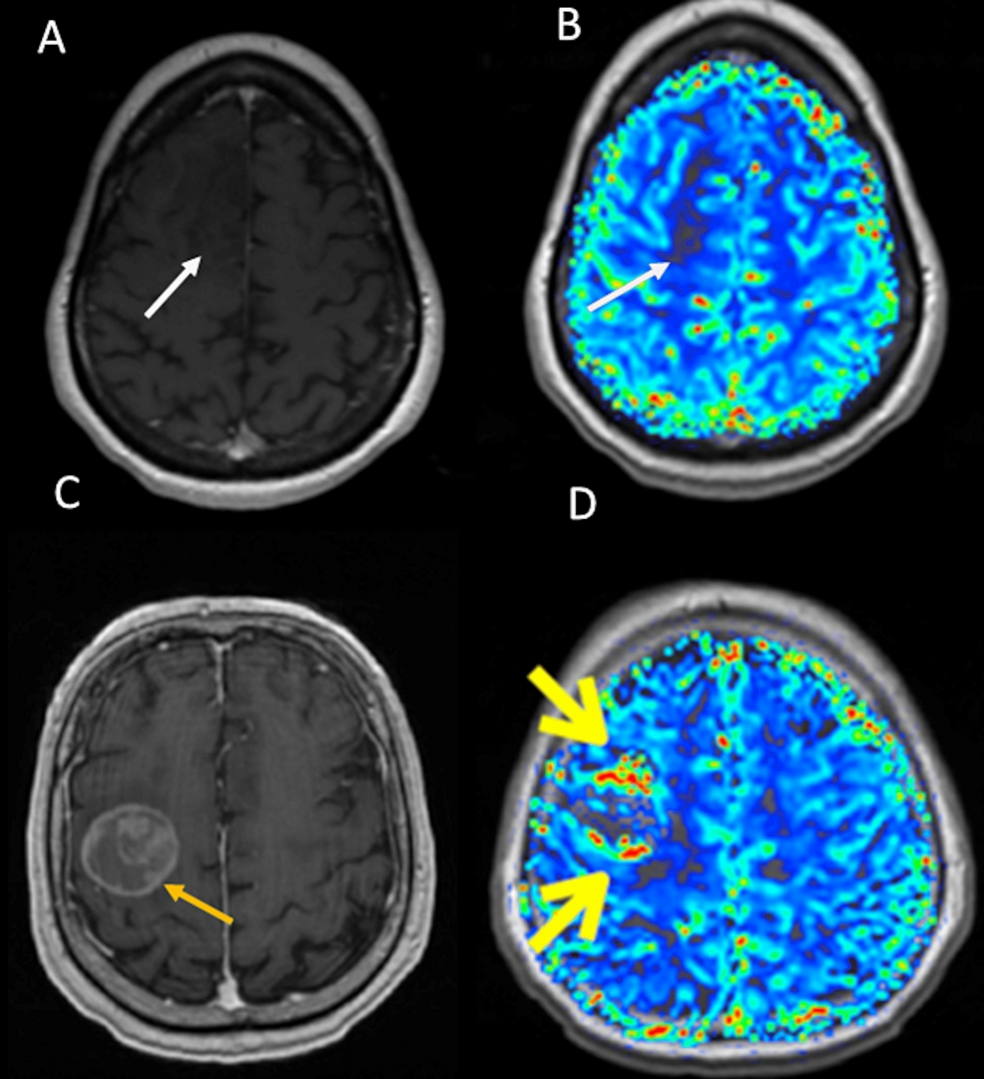
Role of DSC in differentiating between different brain tumors. (A, B) The images represent a right frontal lobe oligodendroglioma (white arrow). (C, D) The images represent right frontal lobe non-small cell lung cancer metastasis (yellow arrow). (A) Post-contrast T1-weighted MRI shows a non-enhancing right frontal lobe lesion. (B) Color-coded rCBV map from DSC data depicting no hyperperfusion. (C) Post-contrast T1-weighted MRI shows a lesion with heterogeneous enhancement. (D) Color-coded rCBV map from DSC data shows hyperperfusion. DSC, dynamic susceptibility contrast; MRI, magnetic resonance imaging; rCBV, relative cerebral blood volume

In contrast to ASL which uses water molecules as endogenous tracers, DCE and DSC MRI techniques use gadolinium particles as exogenous tracers to visualize cerebral blood perfusion (Figure 2) [9,13-15]. Using ASL and DSC, we can calculate many quantitative parameters that reflect changes in cerebral blood perfusion, such as cerebral blood flow (CBF), cerebral blood volume (CBV), and the mean transit time (MTT) which equals CBV divided by CBF and represents the average time needed by gadolinium particles to move through the blood vessel in the lesion of interest [8,12,16]. Relative CBV (rCBV) represents the ratio between the tumoral CBV and the CBV in the contralateral non-tumoral white matter [17,18]. In Figure 2, we present two different brain tumors having different blood perfusion on DSC. This demonstrates that DSC as one of the advanced MRI techniques can depict the physiologic features of the lesion of interest. This also shows the potential role of DSC in differentiating between different tumors by exposing their different physiologic features, such as permeability and vascularity.

**Figure 3:**
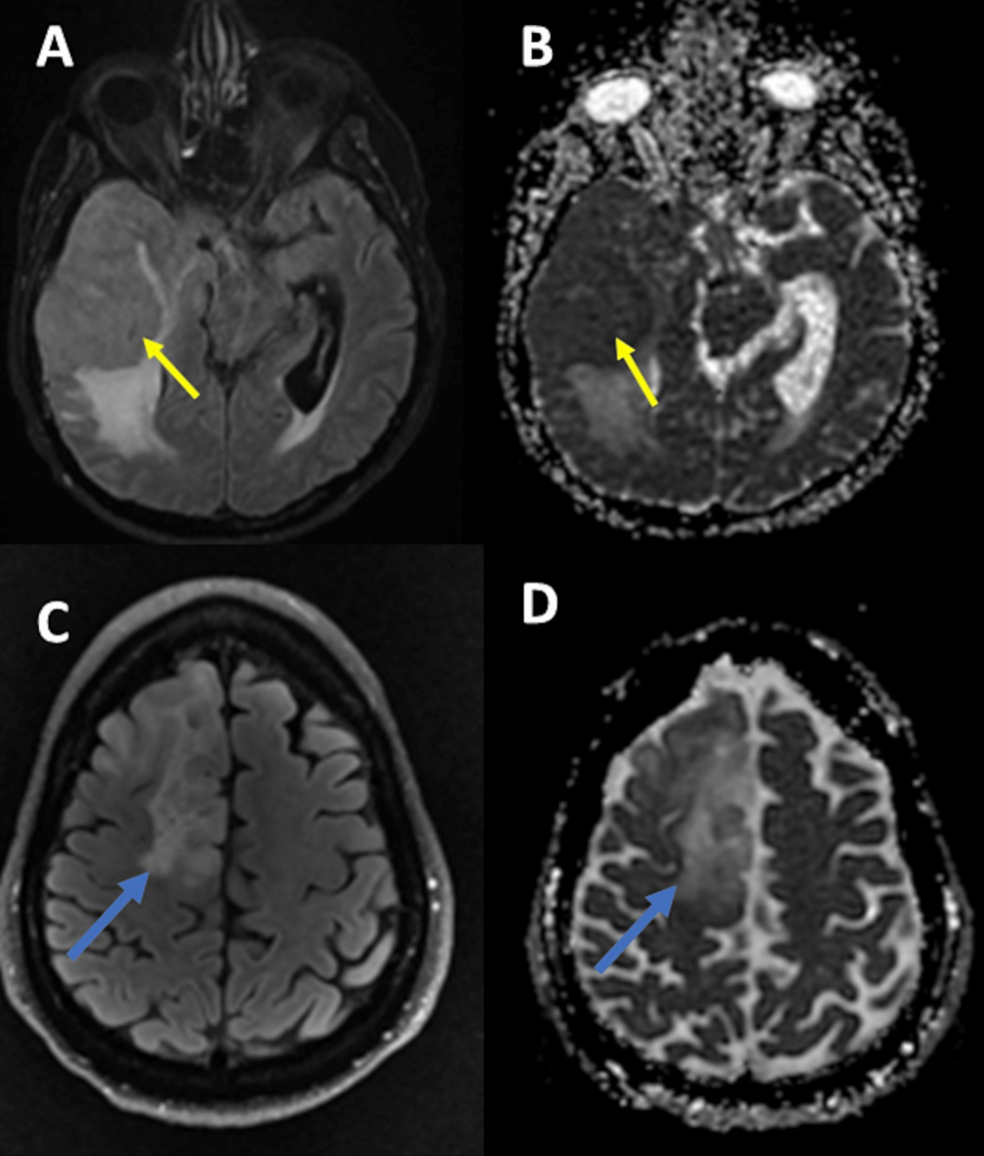
Role of DWI in differentiating between different brain tumors. (A, B) The images represent a right cerebral convexity atypical meningioma  (yellow arrow). (C, D) The images represent a right frontal lobe oligodendroglioma  (blue arrow). (A) FLAIR MRI shows a right cerebral convexity atypical meningioma with ventricular trapping. (B) ADC map from DWI data depicting reduced diffusivity. (C) FLAIR MRI shows a  right frontal lobe oligodendroglioma. (D) ADC map from DWI data shows increased diffusivity (shine-through artifact). DWI, diffusion-weighted imaging; FLAIR, fluid-attenuated inversion recovery; MRI, magnetic resonance imaging; ADC; apparent diffusion coefficient

Using DCE-MRI, we can calculate qualitative, semi-quantitative, and quantitative parameters reflecting cerebral blood perfusion and vascularity. Qualitative parameters include the three time-intensity curve (TIC) patterns. TIC type I represents rapid wash-in followed by the absence of washout with slower wash-in. TIC type II represents rapid wash-in followed by a plateau, while TIC type III demonstrates rapid wash-in followed by slow washout [9,19-21]. Quantitative parameters in DCE quantify the fluid movements between plasma and extravascular extracellular space (EES) and include Vp (fractional plasma volume), Ve (fractional volume of EES per unit tissue volume), Ktrans (forward volume transfer constant between EES and plasma), kep (efflux rate constant). Of interest, Vp reflects vascularity while Ktrans and Ve reflect permeability [13,14,22,23]. Additionally, many semi-quantitative parameters can be measured, such as peak intensity, time to peak, wash-in rate, wash-out rate, time to onset, and area under the curve [9,24]. Water diffusion is visualized in the lesion of interest by diffusion-weighted imaging (DWI). Any change in water diffusion can reflect changes in cellularity or membrane integrity in the tissues of interest. Using DWI, the apparent diffusion coefficient (ADC) can be calculated [10,25,26]. In the case of increased cellularity or fluid viscosity, water diffusion will decrease. As a result, DWI will give higher signal intensity and lower ADC values (Figure 3) [26-28]. In Figure 3, we present two different brain tumors having different diffusion and ADC. Compared to meningioma, oligodendroglioma was found to have increased diffusibility. This indicates that oligodendroglioma has decreased cellularity compared to meningioma. This demonstrates that DWI as one of the advanced MRI techniques can depict the physiologic features of the lesion of interest and differentiate between various tumors. 

## Review

Radiotherapy for brain metastases

Surgical resection, single-fraction stereotactic radiotherapy (SRS), hypofractionated stereotactic radiotherapy (HFSRT), and whole-brain radiotherapy (WBRT) are considered the current treatment options for brain metastases [1,29,30]. Surgical resection may be not useful in surgically inaccessible areas. Additionally, it does not have a significant role in the treatment of multifocal brain metastases. WBRT has been the treatment of choice for multifocal metastases and occult micrometastases. Moreover, It has a significant role in improving quality of life, reducing neurological symptoms, and improving local control. However, cognitive deterioration has been found to be associated with WBRT [30]. In contrast to that, SRS has been shown to have fewer negative cognitive effects. However, SRS has a limited role in the management of multifocal brain metastases [29]. Radiation effect is achieved by its ability to cause DNA damage and induce apoptosis [31]. In contrast, radiation can induce tumor cells’ proliferation, invasiveness, and epithelial-mesenchymal transition (EMT) [32]. Besides developing radioresistance, this paradoxical effect of radiotherapy is considered a challenging problem in the field of radiotherapy. This highlights the importance of developing new modalities to diagnose radioresistance early to avoid any unnecessary exposure to radiation and differentiate between metastases recurrence versus post-radiation changes. Quantitative MRI techniques including DWI, DSC, ASL, and DCE represent potential biomarkers to diagnose metastases recurrence and radioresistance. In this review, we will focus on recent studies discussing the possibility of using DWI, DSC, ASL, and DCE to diagnose radioresistance and recurrence in patients with brain metastases.

Quantitative MRI techniques to assess brain metastases response to radiotherapy

Using DWI and DCE, Shah et al. compared the perfusion and diffusion from pre-radiosurgery and post-radiosurgery brain metastases images. They found that pre-radiosurgery Ve and Ktrans are higher in patients with radioresistance. Using DWI, they found that the early post-radiosurgery perfusion fraction (f) is higher in patients with a progressive course of brain metastasis compared to patients with partial or complete responses to radiotherapy [33]. This suggests the potential role of these radiologic parameters as possible biomarkers to predict the treatment response and radioresistance in patients with brain metastases. Using DWI and DCE, Ye et al. compared the treatment outcomes in patients with brain metastases who were treated with WBRT and gefitinib. They found that ADCpost, ΔADCpost, and tumor regression rate (based on the combined tumor diameter from both DWI and DCE) are significantly different between the treatment-resistant group and the treatment-sensitive group [34]. This highlights the role of DWI and DCE as potential modalities to predict treatment response in patients with brain metastases. In a retrospective study by Zakaria et al., they found that higher post-radiation tumor ADC in patients with brain metastases was significantly associated with prolonged survival [35]. Given that high ADC indicates decreased cellularity and increased apoptosis, high ADC may play a role as a prognostic radiologic biomarker in patients receiving radiotherapy for brain metastases. In another study by Lee et al., they found that high tumor ADC values were seen in controlled brain metastases. In contrast, low tumor ADC values were associated with brain metastases with poor control [36]. Using ASL and DSC, Weber et al. found that the relative regional CBF ratio between the metastatic lesion and grey matter before radiotherapy and at a six-week follow-up can predict the response to radiotherapy. They found that an early relative regional CBF decline can predict tumor remission [37].

Quantitative MRI techniques to differentiate between post-radiation changes or necrosis versus brain metastases recurrence

An enhancing lesion following radiotherapy of brain metastases could be because of post-radiation necrosis or metastases recurrence [38]. Differentiating between these two types of lesions is crucial to avoid unnecessary treatment. While post-radiation changes need serial follow-up MRI, metastases recurrence may need surgery or radiotherapy [39-41]. This highlights the importance of developing rapid non-invasive modalities to differentiate between post-radiation changes and metastases recurrence. Quantitative MRI techniques, such as DWI, DCE, DSC, and ASL have been found as promising modalities to differentiate between tumor recurrence and post-radiation changes. Using DWI, Crowe et al. found that WBRT-treated metastases having significantly increased ADC are positively correlated with apoptotic cells. High ADC indicates decreased cellularity and increased apoptosis and necrotic tissue. Using DCE, They also found that Ktrans (a permeability parameter) is significantly higher in WBRT-treated metastases compared to the control group [42]. This indicates that WBRT disrupts BBB and BTB, subsequently increasing permeability. In another study by Hainc et al., they found that centrally restricted diffusion is associated with radiation-induced necrosis with a sensitivity of 83% and specificity of 64% compared to tumor progression. They concluded that in the case of the absence of centrally restricted diffusion, there is a low probability of radiation-induced necrosis [43]. Measuring interval changes in CBV and Ktrans between pre-and post-radiation brain metastases, Knitter et al. found that interval decrease in CBV and Ktrans is associated with post-radiation changes and can differentiate them from true tumor progression [44]. Similar results were reported by Kuo et al. They found that increased relative CBV can differentiate between tumor recurrence and post-radiation changes in patients with brain metastases [45]. Of interest, Starck et al. found that pre-radiotherapy relative CBV and CBV are significantly decreased in patients with brain metastases who had true progression or early death after radiotherapy [46]. Using DCE and DSC, Morabito et al. found that there is a significant correlation between diagnosing recurrent brain metastases with Ktrans (98% of sensibility and 97% of specificity) and with rCBV (88% of sensitivity and 75% of specificity) [47]. This highlights the possibility of using rCBV and Ktrans as potential biomarkers to differentiate between recurrent brain metastases versus post-radiation changes and necrosis. In a systematic review by Kwee et al., they examined the accuracy of DSC in diagnosing post-radiation metastases recurrence. They found that DSC perfusion results have a pooled sensitivity of 81.6% and specificity of 80.6% to diagnose recurrent brain metastases [48].

## Conclusions

Quantitative MRI techniques including DWI, DSC, ASL, and DCE represent potential biomarkers to diagnose metastases recurrence and radioresistance. This will help in avoiding any unnecessary exposure to radiation and differentiating between metastases recurrence versus post-radiation changes. 
